# Supplementary material for: Quercus glauca Acorn Seed Coat Extract Promotes Wound Re-Epithelialization by Facilitating Fibroblast Migration and Inhibiting Dermal Inflammation
Source: Biology (Basel). 2024 Sep 28;13(10):775. doi: 10.3390/biology13100775 (PMC11505045; doi:10.3390/biology13100775)
Supplement: Supplementary file 1 [file biology-13-00775-s001.zip › biology-3208646-supplementary.pdf]

biology

Article

Quercus Glauca Acorn Seed Coat Extracts Promote Wound Re-epithelialization by Facilitating Fibroblast Migration and Inhibiting Dermal Inflammation

Shin-Hye Kim[1,+], Hye-Lim Shin[1,2,+], Tae Hyun Son[1], So-An Lim[3], Dongsoo Kim[1], Jun-Hyuck Yoon[1], Hyunmo Choi[4], Hwan-Gyu Kim[2] and Sik-Won Choi[1,*]

1 Forest Biomaterials Research Center, National Institute of Forest Science (NIFoS), Jinju 52817, Korea
2 Department of Biological Sciences, Jeonbuk National University, Jeonju 54896, Korea
3 Pharmacogenomics Research Center, Inje University College of Medicine, Busan 47392, Korea.
4 Department of Forest Bioresources, National Institute of Forest Science (NIFoS), Suwon 16631, Korea
\+ These authors contributed equally to this work.
* Correspondence: superwon@korea.kr; Tel.: +82-55-760-5093

Citation: To be added by editorial staff during production.

Academic Editor: Firstname Lastname

Received: date
Revised: date
Accepted: date
Published: 28 September 2024

Copyright: © 2024 by the authors. Licensee MDPI, Basel, Switzerland. This article is an open access article distributed under the terms and conditions of the Creative Commons Attribution (CC BY) license (https://creativecommons.org/licenses/by/4.0/).

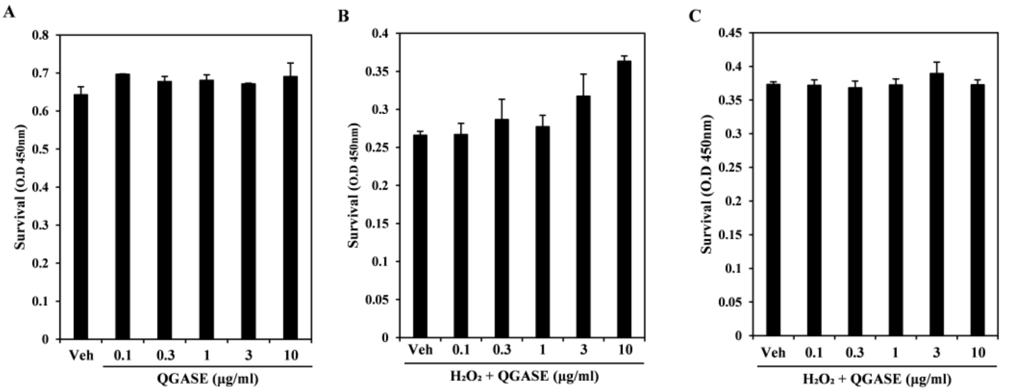

Supplementary Figure S1. The effect of QGASE on the viability of HFs cells was evaluated with the Cell Counting Kit-8 (CCK-8) assay. (a) HF cells were cultured with vehicle or QGASE (0.1, 0.3, 1, 3, and 10 μg/mL) on a 96-well plate to evaluate cell proliferation and cytotoxicity, as illustrated in Figure 1. (b) HF cells were treated with vehicle or QGASE in the presence of hydrogen peroxide (H_2O_2) at a concentration of 8.8 mM in a 96-well plate to investigate cell proliferation and cytotoxicity, as depicted in Figure 3. (c) HF cells were cultured with vehicle or QGASE in the presence of H_2O_2 at a concentration of 500 μM in a 96-well plate to measure cell proliferation and cytotoxicity, as shown in Figure 4.

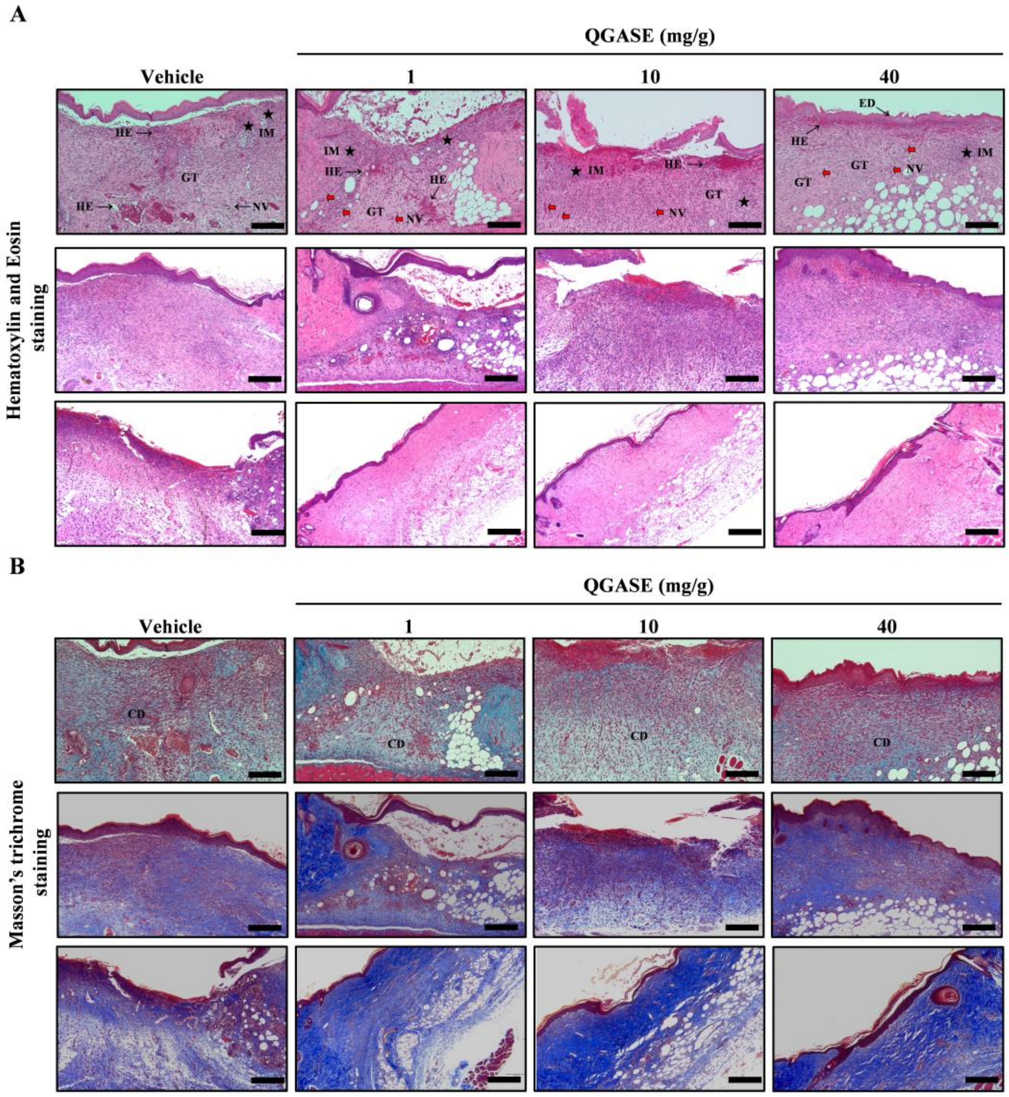

Supplementary Figure S2. The in vivo wound-healing effects of QGASE were demonstrated. (a) Skin tissue samples were fixed in 3.7% formalin for 24 hours and subsequently sectioned to a thickness of 4 μm. The samples were then analyzed using hematoxylin and eosin (H&E) staining. (b) Additionally, Masson's trichrome staining was employed. The following abbreviations are used: GT denotes granulation tissue formation, HE indicates hemorrhage, Thick Black Arrow symbol, IM refers to inflammation, Black Star symbol, NV signifies neovascularization, Red Arrow symbol, ED represents epithelialization, Thin Black Arrow symbol, and CD stands for collagen deposition. scale bar is 200 μm.

Supplementary Figure S3. LC – MS/MS chromatogram of polyphenolic compound

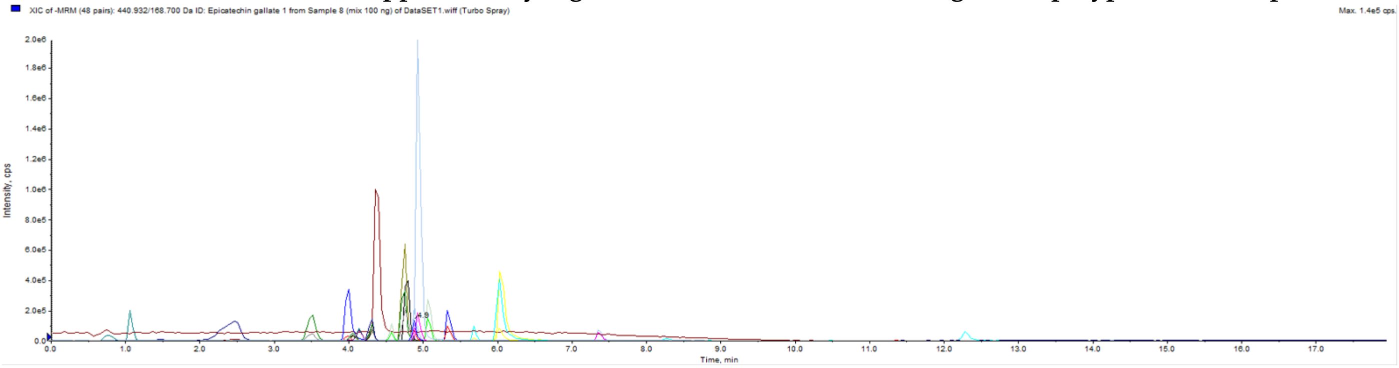

LC – MS/MS chromatogram of 12 standard polyphenol compounds at 100ppb

Disclaimer/Publisher's Note: The statements, opinions and data contained in all publications are solely those of the individual author(s) and contributor(s) and not of MDPI and/or the editor(s). MDPI and/or the editor(s) disclaim responsibility for any injury to people or property resulting from any ideas, methods, instructions or products referred to in the content.
